# Exploring the mechanism of tetramethylpyrazine in the treatment of osteoarthritis based on network pharmacology

**DOI:** 10.3389/fchem.2024.1415390

**Published:** 2024-10-30

**Authors:** Juncen Li, Daiying Song, Bohui Li, Yajie Wang, Huilin Sun, Qinglin Li, Xiangming Lin, Di Wang, Guangdong Zhou, Yu Liu

**Affiliations:** ^1^ College of Clinical Medicine, Shandong Second Medical University, Weifang, China; ^2^ Shanghai Key Laboratory of Tissue Engineering, Department of Plastic and Reconstructive Surgery, Shanghai 9th People’s Hospital, Shanghai Jiao Tong University School of Medicine, Shanghai, China; ^3^ National Tissue Engineering Center of China, Shanghai Jiao Tong University, Shanghai, China; ^4^ Fudan University is affiliated to Shanghai Fifth People’s Hospital, Fudan University, Shanghai, China

**Keywords:** osteoarthritis, network pharmacology, Tetramethylpyrazine, molecular docking simulation, bioinformatics analysis

## Abstract

**Background:**

Osteoarthritis (OA) is the most common joint disease, which mainly damages articular cartilage and involves the whole joint tissue. It has the characteristics of long course, repeated symptoms and high disability rate, and the incidence trend is gradually increasing. Tetramethylpyrazine (TMP) is the main alkaloid active substance in Ligusticum wallichii, a traditional Chinese medicine, which has the effect of promoting blood circulation and dredging collaterals, and has a good effect on the treatment of early OA, but its molecular mechanism has not been fully clarified so far. Based on network pharmacology, molecular docking simulation and animal experiments, this study explored the target and molecular mechanism of TMP in the treatment of OA.

**Methods:**

We used PubChem, SwissTargetPrediction, and PharmMapper databases to predict the molecular structure and potential targets of TMP. GeneCards and DisGeNET databases were used to predict the relevant targets of OA. Apply UniProt database to convert targets into unified gene names, and proofread and remove duplicate gene names. The intersection targets of TMP and OA obtained on venny2.1.0 website were submitted to the STRING database to construct a PPI network. CytoScape 3.8.2 software was used to analyze the PPI network and obtain the sub-network modules and 10 key targets. The intersection targets of TMP and OA were analyzed by Kyoto Encyclopedia of Genes and Genomes (KEGG) and Gene Ontology (GO) enrichment using DAVID 6.8 database. The intersecting targets of TMP and OA, the biological process of GO enrichment, and KEGG signaling pathway were imported into Cytoscape 3.8.2 software to construct the TMP-target-pathway network diagram. Use molecular docking technology to simulate the interaction between TMP molecules and key targets, and predict the binding mode and binding ability. Animal models of rabbit knee osteoarthritis were prepared, and magnetic resonance imager (MRI) and fluorescence quantitative PCR (RT-qPCR) were used to observe the effect of TMP in treating OA as well as the expression of key target genes.

**Results:**

585 potential targets of TMP, 3,857 potential targets of OA, and 49 intersecting targets of TMP and OA were obtained. The top 10 key target genes were obtained, in order of ranking: ALB, ESR1, IL10, CAT, F2, MPO, C3, CYP3A4, CYP2C9, ANXA1. GO and KEGG analysis implied that the key targets might act on OA by affecting endothelial cell permeability, peri-articular microcirculatory status, NETs production, activation of complement system and coagulation pathway, regulation of immune function of macrophages and T cells, and substance metabolism pathway *in vivo*, etc. The molecular mechanism might involve the formation of neutrophil extracellular trap, regulation of the actin cytoskeleton, complement and coagulation cascades, and T cell receptor signaling pathways, etc. Molecular docking simulations showed that the binding energy of IL10 and ANXA1 to TMP was greater than -5Kal/mol, but the other key target proteins showed better binding to TMP, and the binding energy was less than -5 kcal/mol. Animal experiments showed that TMP had a significant therapeutic effect on OA. The TMP group had significantly reduced knee joint effusion and bone marrow damage compared to the OA group (*p* < 0.05). The qRT-PCR results showed that compared with the OA group, the mRNA expression of ESR1, CAT, C3, CYP3A4, CYP2C9, and ANXA1 in the TMP group increased (*p* < 0.05), while there was no significant difference in mRNA expression of ALB, IL-10, F2, MPO, etc. (*p* > 0.05).

**Conclusion:**

TMP is effective in the treatment of OA, with multi-target and multi-pathway interactions. ESR1, CAT, C3, CYP3A4, CYP2C9, and ANXA1 may be potential targets for TMP treatment of OA. The molecular mechanism mainly involves the formation of neutrophil extracellular trap, regulation of the actin cytoskeleton, complement and coagulation cascades, and T cell receptor signaling pathways, etc.

## 1 Introduction

Osteoarthritis (OA) is characterized by a long duration of disease, recurrent symptoms, and a high disability rate and is closely related to age, gender, weight, and occupation. It has become a high-incidence disease, and the incidence trend group has gradually increased (Safiri et al., 2020). According to epidemiological data, the prevalence of OA in China is 50% in people over 60 years of age and 80% in people over 75 years of age, with a disability rate of 53% (Wang et al., 2016), and is expected to become the single most significant cause of disability in the general population by 2030 (Loeser, 2017). OA involves a series of destructive inflammatory processes after onset and is accompanied by the destruction of joint integrity, joint dysfunction, and pain (Aleem et al., 2012). Histopathological manifestations include periarticular bone formation, subchondral bone wear and tear destruction, and synovial changes (Liu and Wang, 2013). According to Chinese medicine, the etiology of OA is mainly based on phlegm and blood stasis blocking collaterals, cold and dampness obstruction, liver and kidney deficiency, and deficiency of Qi and blood, among which “blood stasis” is the main causative factor of the disease and also the pathological product of prolonged failure. Therefore, dispersing blood stasis and dredging collateral is an essential therapeutic measure to prevent and treat OA.

Tetramethylpyrazine (TMP) is the most critical alkaloid active substance in Ligusticum Wallichii that improves blood circulation and disperses stasis and is a novel calcium antagonist that can dilate blood vessels, improve microcirculation in tissues, inhibit platelet adhesion and aggregation in blood vessels, reduce whole blood hyperviscosity and blood cell aggregation index, and exert improved blood rheology (Luo and He, 2018). According to its effect of promoting blood circulation and unblocking collaterals, it is often used to treat the syndrome of qi and blood stasis. In the clinical treatment of early OA, it has shown promising efficacy. It can effectively inhibit the pathological process of articular cartilage (Hu et al., 2006), reduce the inflammatory microenvironment around joints, scavenge oxygen free radicals (Jiang and Wang, 2016), inhibit chondrocyte apoptosis, maintain cartilage phenotype, promote collagen and proteoglycan synthesis, maintain the balance of cartilage matrix synthesis and degradation, protect chondrocytes, and promote articular cartilage repair (Li et al., 2018; Liang et al., 2019; Xie et al., 2018).

Although TMP has achieved good clinical results in the prevention and treatment of OA, the pathogenesis of OA is complex and diverse, and the key targets and molecular mechanisms involved in the treatment of OA with TMP have not yet been fully elucidated.

Here, We predicted potential targets of TMP and OA from PubChem, SwissTargetPrediction, PharmMapper, GeneCards, and DisGeNET databases and obtained the intersection targets of TMP and OA on the venny2.1.0 website. Afterward, the Kyoto Encyclopedia of Genes and Genomes (KEGG) and Gene Ontology (GO) enrichment using the DAVID 6.8 database analyzed the TMP and OA intersection targets. Moreover, the Protein-protein interaction (PPI) network and the top 10 key targets (ALB, ESR1, IL10, CAT, F2, MPO, C3, CYP3A4, CYP2C9, ANXA1)were identified according to degree parameters and clustering coefficient. Furthermore, The intersecting targets of TMP and OA, the biological process of GO enrichment, and the KEGG signaling pathway were imported into Cytoscape 3.8.2 software to construct the TMP-target-pathway network diagram. Moreover, molecular docking technology was used to simulate the interaction between TMP molecules and critical targets and predict the binding mode and binding ability. Animal models of rabbit knee osteoarthritis were prepared, and a magnetic resonance imager (MRI) and fluorescence quantitative PCR (RT-qPCR) were used to observe the effect of TMP in treating OA and the expression of the top 10 target genes. In short, TMP effectively treats OA with multi-target and multi-pathway interactions. ESR1, CAT, C3, CYP3A4, CYP2C9, and ANXA1, among the top 10 key targets, may be potential targets for TMP treatment of OA. The molecular mechanism mainly involves the formation of neutrophil extracellular trap, regulation of the actin cytoskeleton, complement and coagulation cascades, and T cell receptor signaling pathways.

## 2 Materials and methods

### 2.1 Screening of potential targets of TMP and OA

The molecular structure of TMP was searched online using the PubChem (https://pubchem.ncbi.nlm.nih.gov/) database. Using SwissTargetPrediction (http://www.swisstargetprediction.ch/), PharmMapper (http://www.lilab-ecust.cn/pharmmapper/index.html) databases to predict the TMP targets of action, and predicted relevant targets of OA using GeneCards (https://www.genecards.org/), DisGeNET (https://www.disgenet.org/) databases. The UniProt (http://www.uniprot.org/uploadlists/) database was applied to convert targets to uniform gene names. Target genes at the intersection of TMP and OA were obtained using the venny2.1.0 website (https://bioinfogp.cnb.csic.es/tools/venny/).

### 2.2 Construction of protein-protein interaction (PPI) network between intersecting target proteins

The STRING (https://string-db.org/) database was used to collect interaction information of the intersecting target genes and construct the PPI network. The core subnetwork modules in the PPI network were obtained by clustering analysis using the MCODE plug-in in CytoScape 3.8.2 software. The top 10 essential target genes ranked by degree value size were screened using the CytoHubba plug-in.

### 2.3 Functional and pathway enrichment analysis of target genes

The intersecting target genes were analyzed for the Kyoto Encyclopedia of Genes and Genomes (KEGG) and Gene Ontology (GO) enrichment using the DAVID 6.8 (https://david.ncifcrf.gov/) database. Moreover, the GO and KEGG enrichment results were arranged in descending order according to the number of enriched genes and plotted graphically using the Microbiotics (http://www.bioinformatics.com.cn/) online tool.

### 2.4 Construction of TMP-target-pathway network diagram

Extract the enriched genes from the biological processes in GO enrichment, and establish a one-to-one correspondence between each gene and its corresponding biological process. Similarly, establish a one-to-one correspondence between each gene in the KEGG signaling pathway and its corresponding signaling pathway. Then, using Cytoscape 3.8.2 software and genes as links, the above connections are integrated to establish a TMP-target-pathway network diagram. The TMP, targets, biological processes, and signaling pathways were represented as nodes in the network; edges characterized the interrelationships among the nodes.

### 2.5 Molecular docking simulations of TMP with critical targets

A molecular docking technique predicted TMP molecules’ mutual binding mode and binding capacity to critical targets. The crystal structures of the vital target proteins were obtained using the RCSB database (https://www.rcsb.org/) as a template for docking. The 3D design of the loaded TMP was obtained using the PubChem database (https://pubchem.ncbi.nlm.nih.gov/). Moreover, TMP and the top 10 essential target proteins (ALB, ESR1, IL10, CAT, F2, MPO, C3, CYP3A4, CYP2C9, ANXA1) were pre-processed for ligand setups using ChemBio3D Ultra14.0 software, PyMOL software and AutoDock Tools1.5.6 software. Molecular docking simulations of TMPs to essential target proteins were performed using AutoDock Vina software. Moreover, The molecular docking results were then visualized using PyMOL software to show the 3D structures, protein residues, and binding bonds of proteins and small molecules.

### 2.6 Experimental animals and OA model preparation

Nine 4-month-old male New Zealand rabbits weighing 2.5–3.0 kg were purchased from Shanghai Yunde Biotechnology Co., Ltd. The Ethics Committee of Weifang Medical College approved the animal experimental plan.

According to the random number table method, 9 New Zealand rabbits were divided into a standard control group (NC group), a model group (OA group), and a TMP group. Each group consists of 6 samples, and the specific plan is as follows: 6 rabbits will be selected as self controls, with the left leg being the OA group and the right leg being the TMP group. The remaining 3 rabbits are all in the NC group.

Preparation method of osteoarthritis model (He et al., 2019; Kopp et al., 1983; Zhang et al., 2017): Use Zoletil (batch number: 83887905) intramuscular injection anesthesia (dose: 0.5 mL/kg), fix in the supine position, and inject 0.5 mL of 8% papain into the knee joint cavity on the first, fourth, and seventh days of the experiment. Except for the NC group, all other New Zealand rabbits underwent modeling.

Starting from the 8th day of the experiment, drug intervention was conducted. The TMP group was given TMP (concentration: 4%, volume: 0.5 mL) intra-articularly in the knee joint, once every 3 days for 4 weeks of treatment. The NC and OA groups were simultaneously injected with an equal amount of physiological saline into the knee joint cavity.

Sampling plan: The first sampling was conducted on the 7th day after the start of the experiment, with 3 samples from each of the OA and TMP groups. The second sampling was conducted on the 5th week after the start of the experiment, with 6 samples from the NC group and 3 samples from each of the OA and TMP groups. At the same time, sterile syringes were used to extract knee joint fluid from the OA and TMP groups.

### 2.7 Main reagents and instruments

Tetramethylpyrazine hydrochloride for injection (TMP): purchased from Pingguang Pharmaceutical Co., Ltd., production lot number: 202105103; HPLC ≥98%; Papain: Shanghai Yuanye Biotechnology Co., Ltd., production lot number: P08A9B67156; HPLC ≥98%.

Real-time quantitative fluorescence PCR instrument (model: ViiA7; brand: Applied Biosystems AB), micro high-speed centrifuge (model: Mini-14k; brand: cyber Green), vortex oscillator (model: QL-902; brand: Haimen Qilin Bell Instrument Manufacturing Co., Ltd.), constant temperature water bath (model: XMTD-8222; brand: Shanghai Jinghong Experimental Equipment Co., Ltd.); Electric homogenizer (model: F6/F10; brand: FLUKO).

### 2.8 Hematoxylin eosin (HE) staining

Select 5 paraffin sections from each group and sample for HE staining. Firstly, the tissue slices are heated and baked at 60 °C for 3–4 h to fix the sample. Subsequently, the slices need to be dewaxed through xylene treatment, which needs to be repeated three times for 10–15 min each time. Next, the slices will undergo a series of gradient alcohol solutions (100%, 95%, 85%, 75%) for hydration treatment, with each stage lasting 2 min, in order to gradually remove moisture from the sample. Afterwards, the sample needs to be rinsed under flowing water for 5 min to remove any remaining processing solution. Subsequently, hematoxylin was used to stain the nucleus, which lasted for 15–20 min. After dyeing, rinse the sample again in flowing water for 1 min to remove excess staining agent. Then, the sample was briefly (for 3 s) immersed in a 1% hydrochloric acid alcohol solution to differentiate the color, followed by a 45 min blueing treatment with flowing water to enhance the staining effect. Finally, the sample was stained with acidified eosin ethanol solution for 2 min to compare the staining details. Afterwards, the slices need to be dehydrated using alcohol solutions with opposite gradients (75%, 85%, 95%, 100%). Next, the sample will undergo three xylene treatments, each lasting 10 min, to achieve transparency. Finally, use neutral resin for sealing and observe the staining results under a microscope to evaluate the quality of staining and the structural details of the sample. Randomly select 5 fields of view for each slice, observe and take photos.

### 2.9 Safranine O staining

Select 5 paraffin sections from each group and sample for Safranine O staining. Heat the tissue slices at 60 °C and bake for 3–4 h to fix the sample. Subsequently, the slices were dewaxed using xylene, repeated three times for 10–15 min each time. Next, the slices will be subjected to a gradient alcohol solution (100%, 95%, 85%, 75%) for hydration treatment, with each stage lasting for 2 min, in order to gradually remove moisture from the sample. Afterwards, the sample needs to be rinsed under flowing water for 5 min to remove any remaining processing solution. Soak with hematoxylin for 15–20 min, rinse with running water for 1 min, differentiate with 1% hydrochloric acid alcohol for 3 s, return to blue with running water for 45 min, stain with safranin solution for 30 min, and wash with anhydrous ethanol three times. Differentiate with 1% hydrochloric acid alcohol for 3 s, then use xylene transparent again, encapsulate the slices with neutral resin, observe the results under a microscope, and scan and collect images. Randomly select 5 fields of view for each slice, observe and take photos.

### 2.10 Magnetic resonance imaging (MRI)

MRI was obtained by scanning the rabbit knee joint using a magnetic resonance imager (MAGNETOM Skyra 3.0T, Siemens, Germany) at weeks 1 and 5 after the start of the experiment. MRI changes in the knee were evaluated using the scoring system of Bouchgua et al. (2008). The evaluation criteria were as follows: knee effusion score, from 0 to 3 (normal to severe); severity score of knee osteoarthrosis, from 0 to 3 (normal to severe); grading assessment of bone marrow lesions (BMLs): grade 0, no BMLs; grade 1, lesion involving <25% of the chamber volume; grade 2, lesion involving 25%–50%; grade 3, lesion involving >50%. A blinded method was used, and the imaging physicians were asked to evaluate the MRI.

### 2.11 Real-time fluorescence quantitative PCR (RT-qPCR)

Perform RT qPCR detection on the knee joint fluid of OA group and TMP group. RT-qPCR was used to detect the expression of the top 10 essential genes. Total RNA was extracted with TRIzol reagent (Invitrogen). The extracted mRNA was reverse transcribed using the MMLV system from Promega to obtain cDNA. Real-time PCR experiments were performed using cDNA as a template. The reaction system was prepared according to the instructions of the dye method PCR mTMPer mix kit (TaKaRa). The reaction conditions were: 95°C for 1 min (preheating) - 95°C for 15 s - 60°C for 30 s (40 cycles) - solubility curve (95°C for 15 s - 60°C for 1 min - 95°C for 15 s). The relative expression levels of each target mRNA were calculated by the 2^−ΔΔCt^ method. The primer information of each gene was designed by Dexuan Biologicals and synthesized by Invitrogen Company, as shown in Table 1.

**TABLE 1:** Primer information.

Gene name	Forward primer	Reverse primer
ALB	5′GCT TAT TCC AGG GGT GTG T 3′	5′TGC TTT TGC CAA GTC TGT TAC 3′
ESR1	5′ACC AGG GGA AAT GTG TAG A 3′	5′GTG CTG GAC AGA AAT GTG TA 3′
IL10	5′CTG TGG GAT TTG AGT GTC TTA 3′	5′TCG GCT TAG GAG TTA GAA AGT 3′
CAT	5′CTC ACT TTG ACC GAG AGA GAA 3′	5′GCC TTG CTG TAT CTG GTA ATG 3′
F2	5′TGG CAA GTA TGG CTT CTA CAC 3′	5′TGA CTG GGT TGG TTT CTG TAA 3′
MPO	5′CCG TGT CCA AGA ACA ACA T 3′	5′AAG CCA GGT CCA AAA CAG 3′
C3	5′GCA GGA GGC TAG AGA GAT TT 3′	5′AGT CCC TTG ATT TGT TGA TG 3′
CYP3A4	5′TTG CTG TCT CCA ACC TTC AC 3′	5′TTT TCA CCA ACA CAT CTC CAT AC 3′
CYP2C9	5′GCA AAT CCT TCA CCA AAC TGT 3′	5′GAA AAC TCT TCT CCC AGA TCA AC 3′
ANXA1	5′CTA TCT CCA TCT TCG CAG AGT 3′	5′ATT TCT CAA TGT CAC CTT TCA G 3′
b-actin	5′GCA GAA ACG AGA CGA GAT TG 3′	5′GCA GAA CTT TGG GGA CTT TG 3′

### 2.12 Statistical analysis

SPSS23.0 statistical software was used for analysis, and the data were expressed as mean ± standard deviation (x ± s); if the variances were equal, a *t*-test for independent samples was used for comparison between two groups, and One-way ANOVA was used for comparison between multiple groups; if the variances were not equal, the rank-sum test was used, and the test level was α = 0.05.

## 3 Results

### 3.1 Related targets of TMP and OA

**FIGURE 1:**
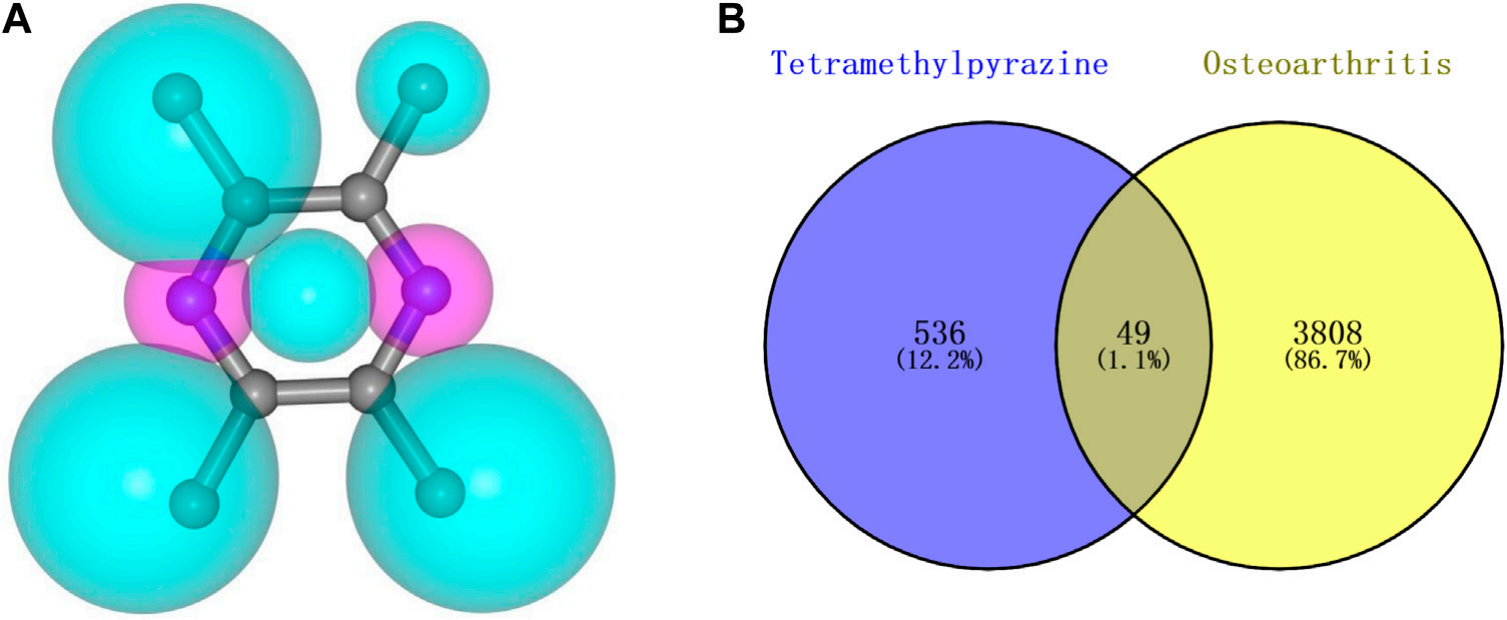
Molecular structure of TMP and intersection targets with OA. **(A)** TMP pharmacophore model and molecular characteristics. **(B)** Intersection of TMP and OA targets.

The pharmacophore model and molecular characteristics of TMP(Figure 1A) and corresponding 585 potential targets were obtained from the SwissTargetPrediction website and PharmMapper database. 3857 OA-related targets were obtained from GeneCards and DisGeNET databases (Figure 1B). Subsequently, the intersection of related marks of TMP and OA was analyzed using Draw Venn Diagram online tools (https://bioinfogp.cnb.csic.es/tools/venny/). As shown in Figure 1B, 49 common targets for TMP and OA were identified.

### 3.2 PPI network and critical target gene analysis

**FIGURE 2:**
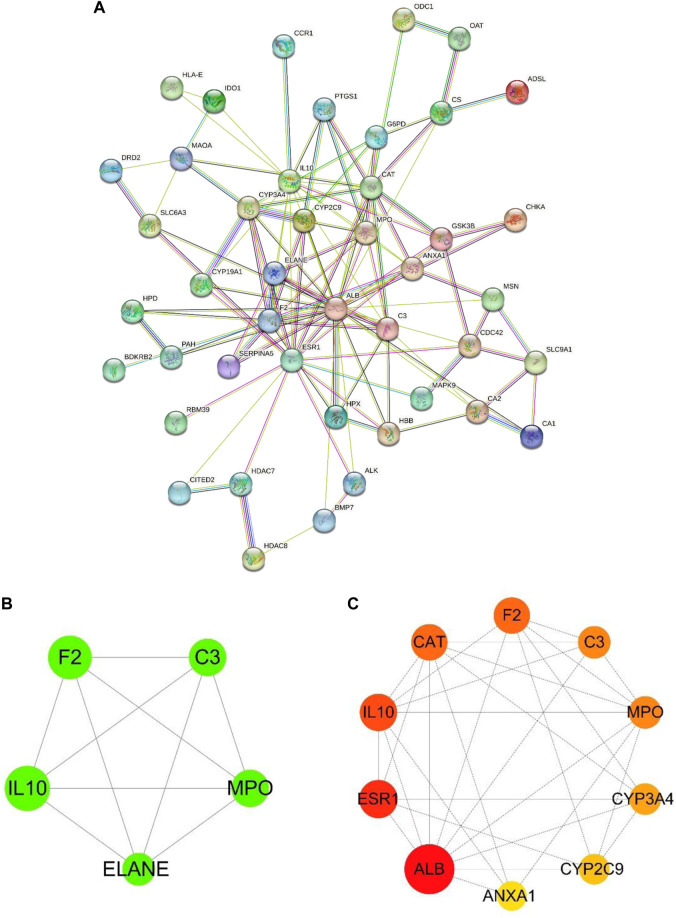
PPI network and critical target genes. **(A)** PPI network of TMP and OA intersection targets. **(B)** The core sub-networks in the PPI network. The node’s size is set according to the node degree value; the more significant the node, the more critical the node. The thickness of the edges is set according to the combination score, and the thicker the border, the stronger the PPI relationship. **(C)** The top 10 essential target genes in the intersection target ranked by degree value; the more significant the node, the more red the color, and the higher the ranking.

Forty-nine common targets for TMP and OA were submitted to the STRING database; the species was selected as “*Homo sapiens*,” the clustering method was set as “K-means clustering,” and then the PPI network was obtained (Figure 2A). Next, the data file downloaded from STRING analysis was visualized utilizing Cytoscape 3.8.2 software. The MCODE plug-in in CytoScape software was used to cluster the interactions and obtain the sub-networks with the highest scores in order of score (Figure 2B). The cytoHubba plug-in was applied to get the top 10 critical genes in order of degree (Figure 2C; Table 2), which were: Albumin (ALB), Estrogen Receptor 1 (ESR1), Interleukin 10 (IL10), Catalase (CAT), Coagulation Factor II (F2), Myeloperoxidase (MPO), Complement C3 (C3), Cytochrome P450 Family 3 Subfamily A Member 4 (CYP3A4), Cytochrome P450 Family 2 Subfamily C Member 9 (CYP2C9), Annexin A1 (ANXA1). The degree value algorithm is based on the degree of a node, which is the number of connections between nodes. The higher the degree of a node, the more connections it has in the network and is therefore considered a more important node.

**TABLE 2:** Docking Binding energy and potential binding site between TMP and core targets.

Targets	Degree	PDB ID	Amino acid residues	Binding energy/Kcal⋅mol^-1^
CYP3A4	8	6BD7	phe-108, arg-106, ile-50	−6.1
CAT	13	1DGB	his-75, thr-361, phe-334	−5.8
MPO	9	1CXP	asp-94, gln-91, gly-90	−5.7
ALB	27	1E7A	leu-453, asn-391	−5.4
ESR1	15	3OS8	ala-350, glu-353, arg-394	−5.2
C3	9	1GHQ	pro-59, ser-110, glu-41	−5.2
CYP2C9	7	5A5I	ala-477, ile-205, leu-361	−5.2
F2	13	3PO1	ser-235, ala-230, glu-232	−5.1
IL10	14	2ILK	Phe-128, gly-135	−4.4
ANXA1	6	1QlS	thr-49, asn-42	−4

### 3.3 Functional enrichment analysis of intersection target genes

**FIGURE 3:**
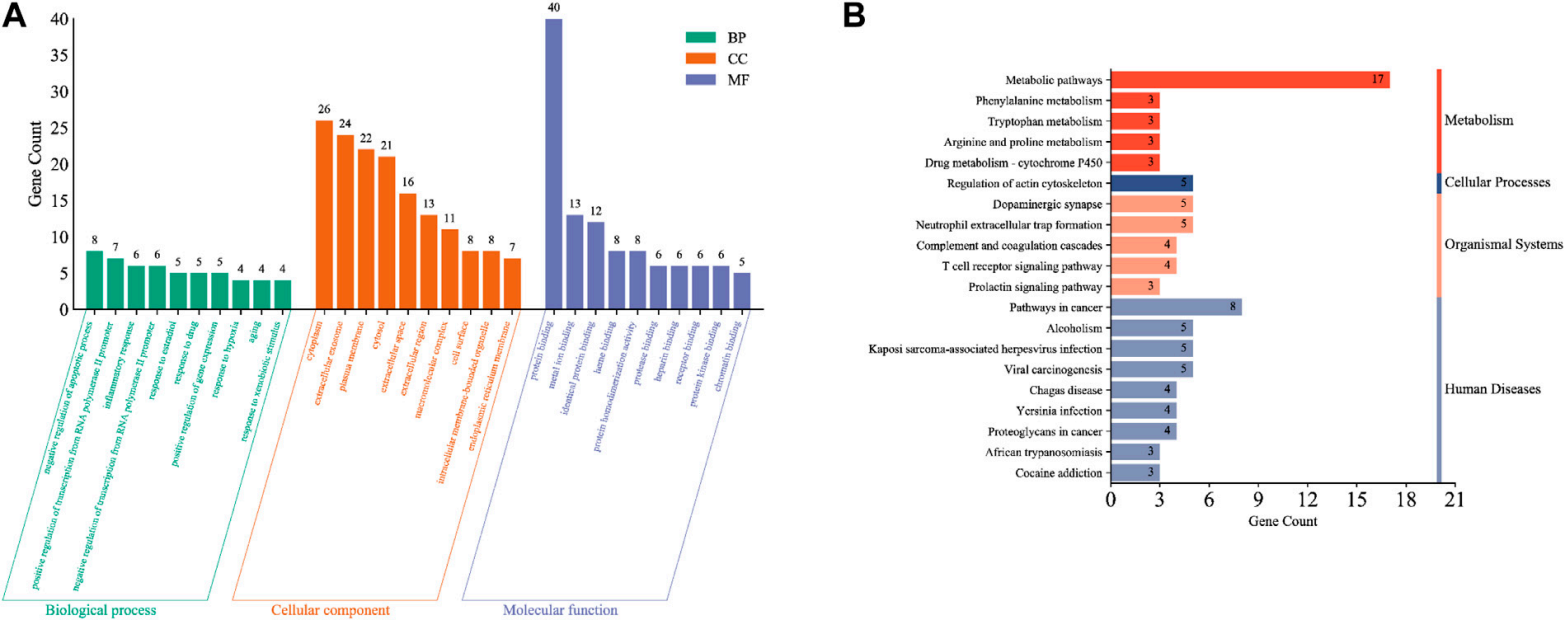
Functional enrichment analysis of intersection target genes. **(A)** Results of GO enrichment analysis of intersection targets. **(B)** Results of KEGG enrichment analysis of cross-target.

GO analysis was used to annotate gene functions and standardize the descriptions of the intersecting target genes according to the biological process (BP), cellular component (CC), and molecular function (MF) categories. We obtained 142 total annotation entries for GO enrichment, including 85 entries for BP, 20 for CC, and 37 for MF, which were arranged in descending order according to the number of enrichment genes. Each category selected the top 10 entries to draw a Bar chart (Figure 3A). From the GO analysis, we found that the intersecting target genes are mainly involved in biological processes such as negative regulation of the apoptotic process, positive regulation of RNA polymerase two promoter transcription, inflammatory response, negative regulation of RNA polymerase two promoter transcription, response to estradiol, drug response, regulation of gene expression, hypoxic response, and aging. Most of the core targets are located in the cytoplasm, extracellular exosomes, plasma membrane, cytoplasm, extracellular space, extracellular compartments, polymer complexes, cell surfaces, cell membrane-bound organelles, and endoplasmic reticulum membranes with molecular functions for binding to proteins, metal ions, hemoglobin, proteases, heparin, receptors, chromatin, and so on.

The KEGG database, which enables pathway analysis of the intersecting target genes to identify biological functions, is divided into the following six classifications: cellular processing, environmental information processing, genetic information processing, human diseases, metabolism, and organismal systems. From the KEGG database, we obtained 23 signaling pathways, which were arranged in descending order according to the number of enriched genes, and the top 20 entries were plotted as a bar chart (Figure 3B). Comprehensive analysis of the KEGG classification results for the intersecting target genes showed enrichment mainly occurred in four classifications: metabolism, cellular processes, organizational systems, and human disease. Moreover, KEGG pathway enrichment analysis suggested that the intersecting target genes were enriched mainly in metabolic pathways, cancer pathways, neutrophil outer trap formation, actin cytoskeleton regulation, complement and coagulation cascade, T cell receptor signaling pathway, prolactin signaling pathway, drug metabolism-cytochrome P450 signaling pathways, etc.

### 3.4 Construction of drug-target-pathway network

**FIGURE 4:**
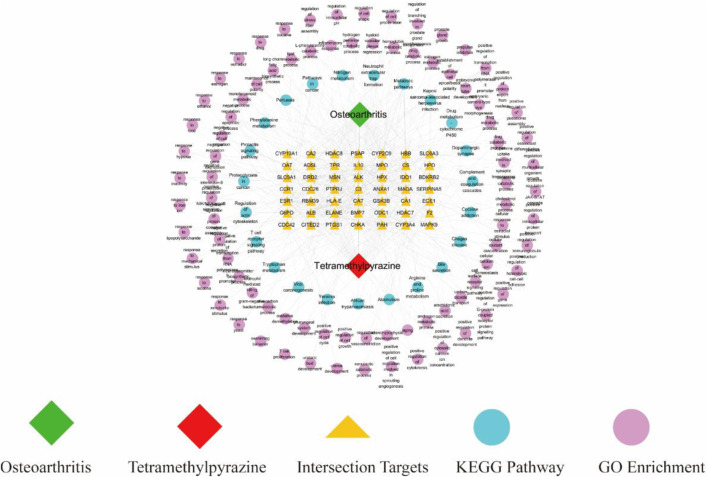
Drug-target-pathway network of TMP for OA.

We drew a drug-target-pathway network diagram based on TMP, 49 intersecting target genes, 85 GO-enriched biological processes, and 23 KEGG-enriched pathways using Cytoscape 3.8.2 software (Figure 4). This diagram suggested that the potential mechanism of TMP in treating OA has the characteristics of the multi-target and multi-pathway interactions.

### 3.5 Molecular docking simulation

**FIGURE 5:**
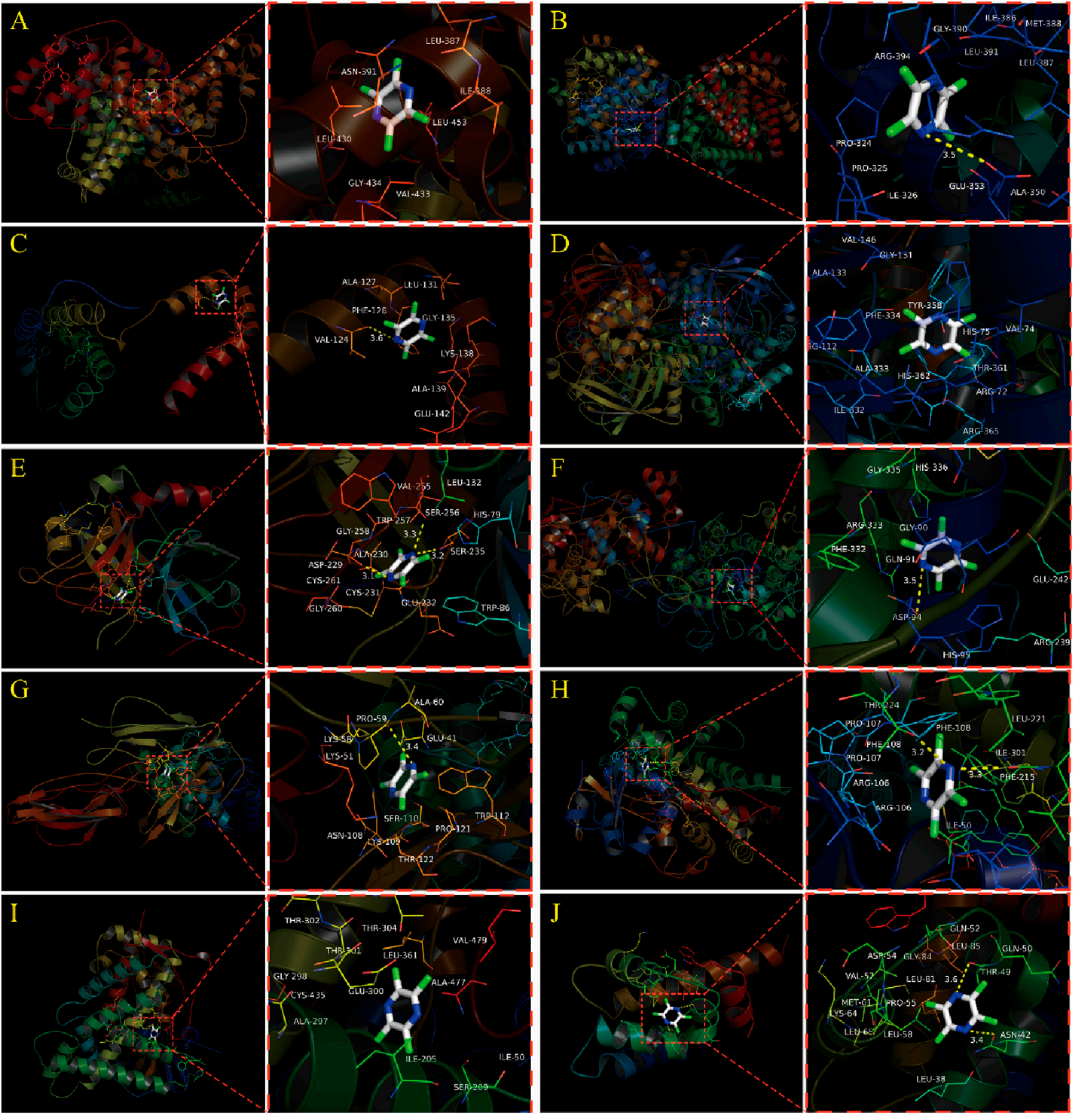
Molecular docking simulation between TMP and Top 10 essential target proteins. **(A)** TMP and ALB. **(B)** TMP and ESR1. **(C)** TMP and IL10. **(D)** TMP and CAT. **(E)** TMP and F2. **(F)** TMP and MPO. **(G)** TMP and C3. **(H)** TMP and CYP3A4. **(I)** TMP and CYP2C9. **(J)** TMP and ANXA1.

The results of this molecular docking showed that the binding energies of other essential target proteins with TMP are less than −5 kcal/mol, except for IL-10 and ANXA1. Moreover, TMP showed good binding to some amino acid residues of crucial target proteins (Table 2), suggesting that TMP has high affinity and activity with OA-related targets (Figures 5A–J).

### 3.6 Histological staining results

**FIGURE 6:**
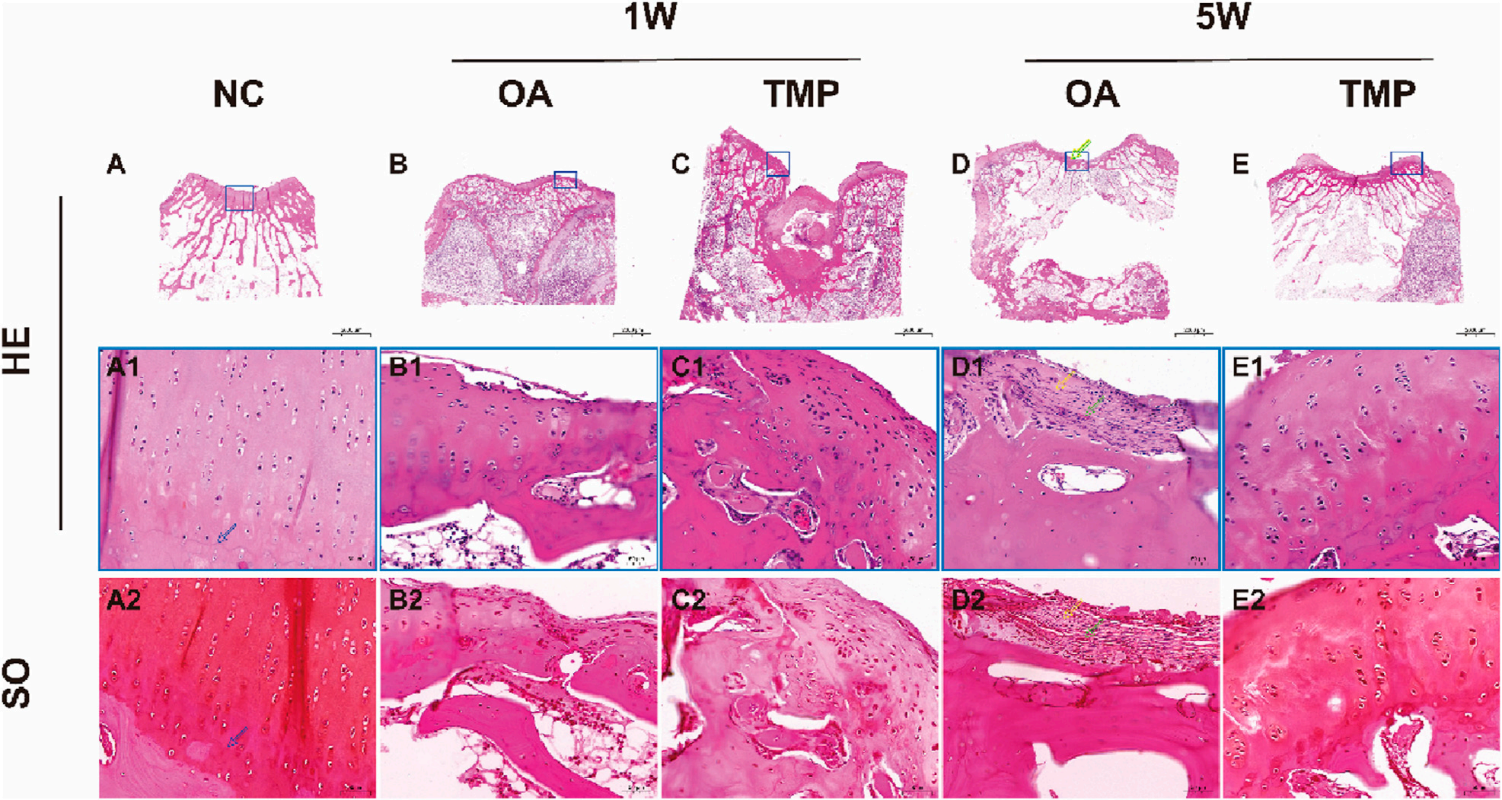
Histological staining results. **NC**: regular control group **(A)**; **OA**: untreated papain-induced osteoarthritis group **(B, D)**; **TMP**: TMP-treated papaininduced osteoarthritis group (Before TMP-treated: **(C)**, After TMP-treated for 4 weeks: **(E)**. HE: Hematoxylin eosin staining. SO: SafranineOStaining. Enlarged image of the blue boxed area in **(A1, A2)**; Enlarged image of the blue boxed area in **(B1, B2)**; Enlarged image of the blue boxed area in **(C1, C2)**; Enlarged image of the blue boxed area in **(D1, D2)**; Enlarged image of the blue boxed area in **(E1, E2)**; The tidemark was marked by **blue arrow**, The collagenous fiber was marked by **yellow arrow**, The blood vessel was marked by **green arrow**.

The HE staining results of the NC group showed that squamous chondrocytes were visible on the surface of the cartilage, with collagen fibers arranged parallel to the joint surface. The chondrocytes in the middle area were circular and arranged diagonally with collagen fibers. There were vertical chondrocyte columns in the deep area, and collagen fibers were perpendicular to the joint surface tissue. A wavy alkaline tide line can be seen between the calcified and uncalcified areas of cartilage, complete and continuous, as shown by the blue arrow in Figures 6A1, A2. Cortical bone can be seen in the subchondral layer, with small pores and vascular pathways inside, containing osteoblasts and osteoclasts. Subchondral trabecular bone presents as randomly oriented bone trabeculae, with hematopoietic stem cells, mesenchymal stem cells, and adipose tissue all deposited in the trabecular foramen. On the sections stained with safranin O, it was clearly observed that proteoglycans were stained shallower in the superficial layer of cartilage, and gradually deepened in the middle and deep regions (Figure 6A2).

After 1 week of modeling, the surface layer of knee joint cartilage in both OA and TMP groups was damaged. Microscopically, the continuity of the cartilage surface layer was interrupted, reaching a depth of up to the middle area of the cartilage. The arrangement of chondrocytes was disordered, and a large number of fibroblasts were observed in the damaged area, secreting a large amount of collagen protein (Figures 6B, C).

After 5 weeks of modeling, the degree of cartilage damage in the OA group significantly increased, with the depth of the damage reaching the deep area of the cartilage, and some even breaking through the calcified area of the cartilage to reach the subchondral bone cortex. The tidal line was almost invisible. Scar formation can be seen in the area of cartilage injury, with blood vessels distributed within it (as indicated by the green arrow). A large number of inflammatory cells and fibroblasts gather, and collagen fibers are arranged in a staggered manner (as indicated by the yellow arrow) (Figures 6D1, D2). The cartilage injury in the TMP group did not worsen progressively, and the degree of injury was significantly reduced (Figure 6E). The surface layer of cartilage shows continuous and orderly arrangement of squamous chondrocytes, with collagen fibers distributed parallel to the joint surface. In the middle and deep regions of the cartilage, circular chondrocytes can be seen arranged in clusters and columns. The tidal line structure between the calcified and non calcified areas of the cartilage is obvious and continuous (Figure 6E1). The results of safranin O staining show a clear boundary between the cartilage and subchondral bone, and the extracellular matrix of the chondrocytes is stained with saffron. The color of the middle and deep regions of the cartilage is darker than that of the lighter layers (Figure 6E2).

### 3.7 MRI examination results

**FIGURE 7:**
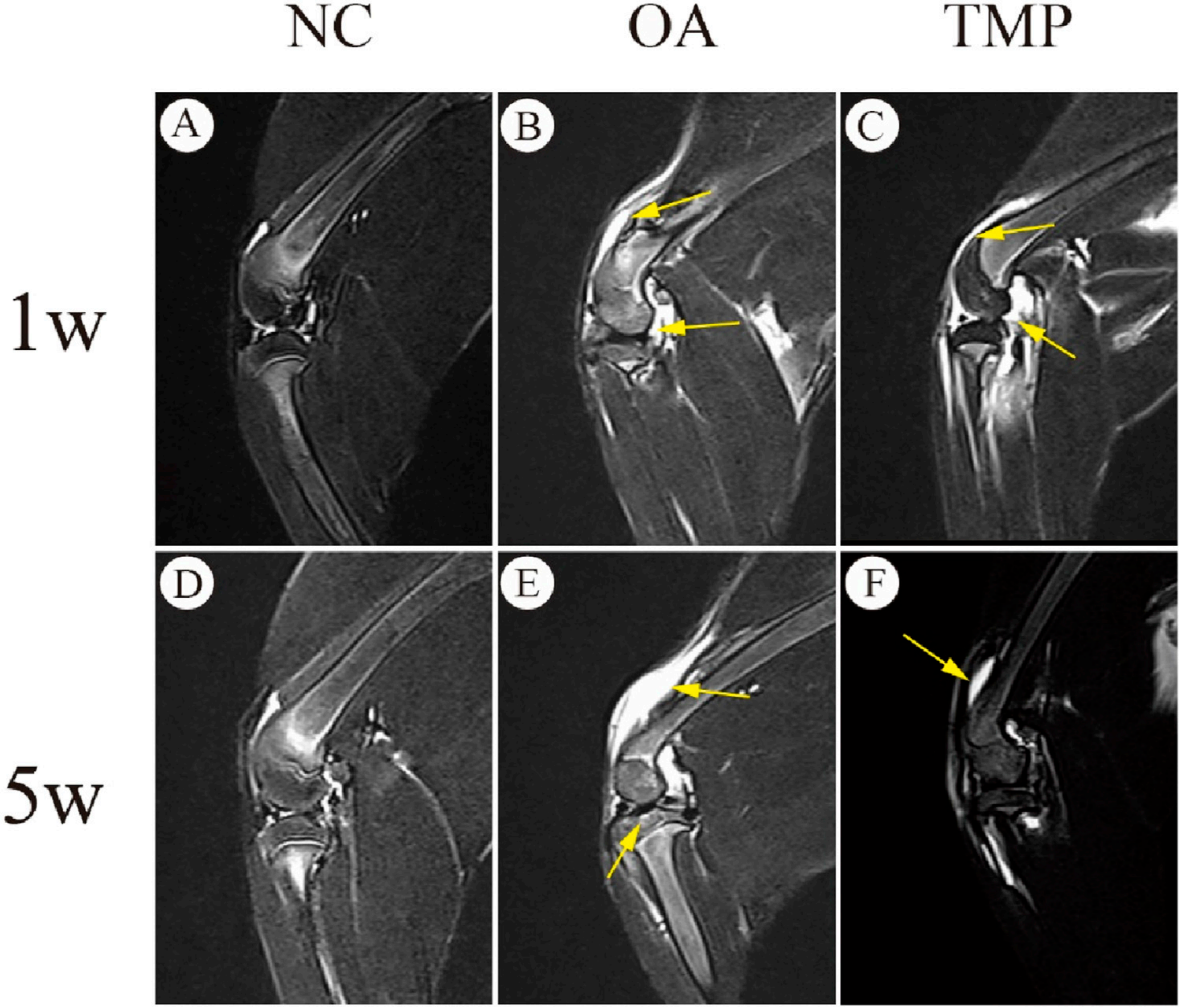
Evaluation of therapeutic efficacy of TMP by MRI images. **NC**: regular control group **(A, D)**; **OA**: untreated papain-induced osteoarthritis group **(B, E)**; **TMP**: TMP- treated papain-induced osteoarthritis group (Before TMP - treated: **(C)**, After TMP - treated for 4 weeks: **(F)**. Joint effusion and bone marrow lesions were (marked by yellow arrow) present in OA and before TMP-treated **(B, C)**. These alterations were improved after TMP treatment for 4 weeks**(F)**. n = 6.

At week one after the start of the experiment (before TMP intervention), knee effusion and bone marrow injury were observed in both the OA and TMP groups (Figures 7B, C), and there was no statistical difference in the scoring results between the two groups (*p* > 0.05) (Table 3). At week five after the start of the experiment (week 4 of TMP treatment), knee effusion was significantly reduced in the TMP group. The degree of bone marrow damage was reduced considerably (Figures 7E, F), which was statistically quite different compared with the OA group (*p* < 0.05) (Table 3). Knee osteochondrosis was not observed in any of the groups in this trial (Figure 7).

**TABLE 3:** MRI scoring results.

	Joint effusion		Bone marrow lesion	
	1w	5w	1w	5w
NC	0.00 ± 0.000	0.00 ± 0.000	0.00 ± 0.000	0.00 ± 0.000
OA	2.00 ± 0.632^ΔΔ^	2.83 ± 0.408^ΔΔ^	1.33 ± 1.032^Δ^	2.50 ± 0.837^ΔΔ^
TMP	1.83 ± 0.752^Δ^	0.50 ± 0.548*	1.67 ± 0.516^ΔΔ^	0.33 ± 0.516*

**Note:** Compared with the regular group.

^Δ^P< 0.05.

^ΔΔ^P< 0.01, TMP, compared to OA group, *p < 0.05.

### 3.8 QRT-PCR analysis results

**FIGURE 8:**
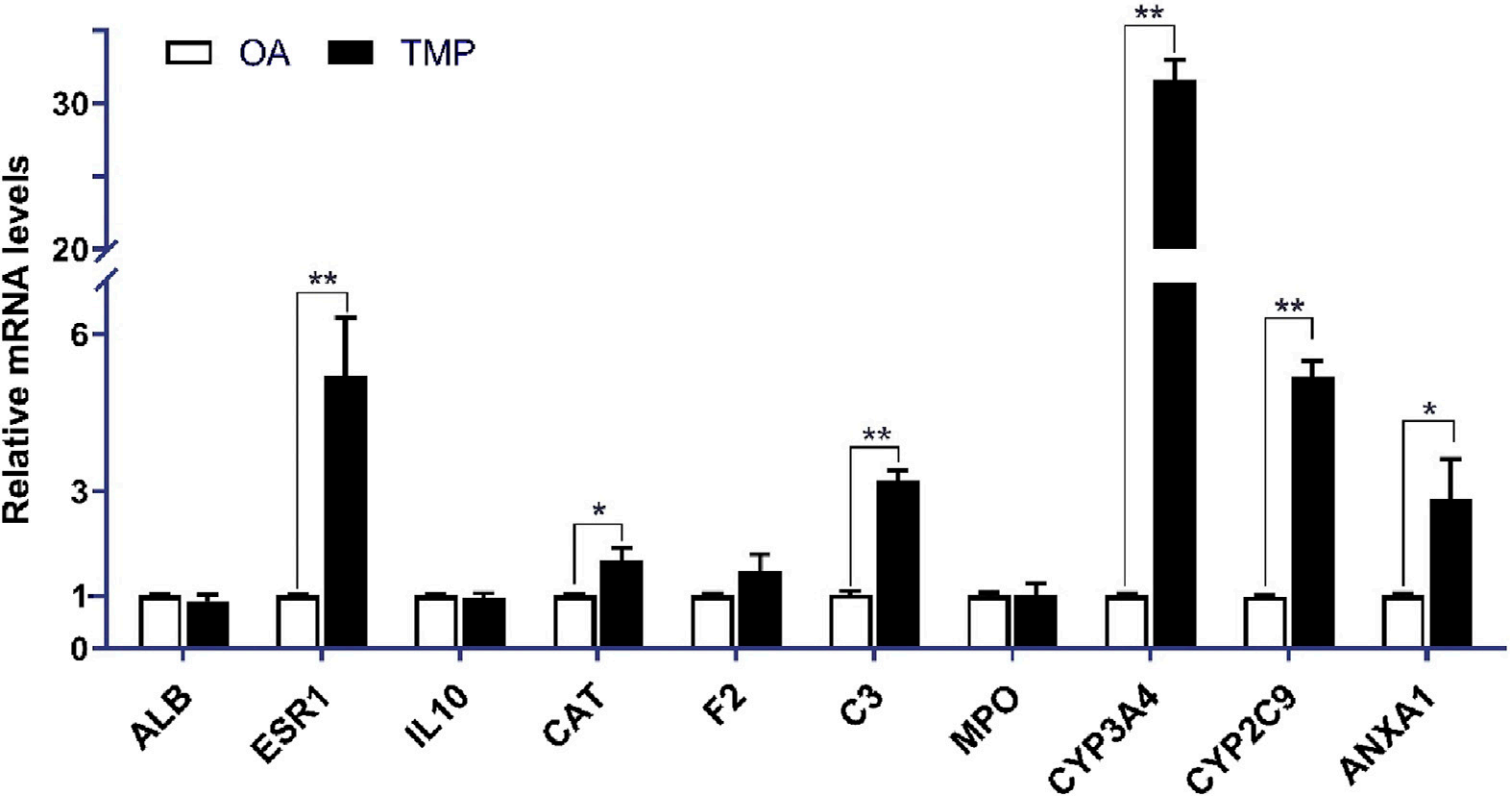
Relative expression of essential target genes in the joint fluid of TMP and OA groups in the 5th week after the experiment. Compared with the OA group, *p < 0.05, **p < 0.01.

Compared with the OA group, the mRNA expressions of ESR1, CAT, C3, CYP3A4, CYP2C9, and ANXA1 were increased in the TMP group (*p* < 0.05), and the mRNA expressions of ALB, IL-10, F2, MPO, were not significantly different (*p* > 0.05) (Figure 8).

## 4 Discussion

### 4.1 Exploration of preparation standards for OA models

This time, rabbits were used as model animals, and their joint structure is easy to operate in experiments. In the experiment, injecting 8% papain into the joint cavity can cause rapid occurrence of osteoarthritis. The modeling time is short, the repeatability is high, the experimental operation is relatively simple, the animal trauma is small, and the obtained model is relatively stable with obvious cartilage damage, which is convenient for histological and imaging observation. The animal model made with papain is similar to the pathological changes of early human OA, and the degree of degenerative changes in joint cartilage is positively correlated with papain concentration and induction time, which can be used to study the mechanism of OA lesions and intervention therapy in different disease cycles.

### 4.2 Analysis of critical targets of TMP for the treatment of OA

We screened ten potential core targets of TMP associated with OA by network pharmacology screening: ALB, ESR1, IL10, CAT, F2, C3, MPO, CYP3A4, CYP2C9, and ANXA1, and the results of molecular docking simulation also showed that TMP could present better binding with most of the potential core targets, with binding energies of less than -5Kcal/mol. However, TMP is a building block molecule of MW 136.19, with extensive pharmacological effects in preclinical models. Therefore, potential interaction with molecular targets is theoretically of low potency and non-selective. In this experiment, we found that only the target genes of ESR1, CAT, C3, CYP3A4, CYP2C9, and ANXA1, whose mRNA expression was increased in the TMP group (*p* < 0.05), showed statistically significant differences between the TMP group and OA group. This suggests that ESR1, CAT, C3, CYP3A4, CYP2C9, and ANXA1 are most likely to be the pharmacological basis of TMP in treating OA.

In human articular chondrocytes, ESR binding to estradiol can inhibit NF-κB pathway activation and thus exert anti-inflammatory effects (Ushiyama et al., 1999; Zhang et al., 2016). CAT is an essential member of the antioxidant enzyme system in living organisms, which can break down H_2_O_2_ into H_2_O and O_2_, preventing the toxic effects of excessive H_2_O_2_ on cells (Oscar, 1900). C3 is a central component of the complement system, which interacts with tumor necrosis factor α (TNF-α), platelet-activating factor (PAF), interleukin 1 (IL-1), interleukin 6 (IL-6), and other factors. C3 is a central component of the complement system, which can synergize with or constrain various cytokines, such as tumor necrosis factor α (TNF-α), platelet-activating factor (PAF), interleukin 1 (IL-1), interleukin 6 (IL-6), and interleukin 8 (IL-8), and plays a vital role in inflammatory response, tissue and organogenesis, modulation of host immune function, mediation of apoptotic cell clearance, and facilitation of tissue repair after injury (Damgaard et al., 2015; Hajishengallis et al., 2019). There is also a close relationship between C3 and bone metabolism (Yu et al., 2021), and C3 can influence osteoclast differentiation through the OPG/RANKL/RANK signaling pathway (Whitmore and Lamont, 2014). CYP3A4 and CYP2C9 are members of the cytochrome P450 enzyme family, which can catalyze the synthesis of steroid hormones, regulate the metabolism of liposoluble hormones, and regulate the metabolism of therapeutic drugs (Bao, 2019; Bulutoglu et al., 2019; Li, 2015). ANXA1 (membrane-associated protein A1) can bind reversibly to phospholipid membranes and calcium ions and can inhibit the migration and adhesion of inflammatory cells (Galvão et al., 2017), inhibit the synthesis and release of inflammatory mediators (Liao et al., 2017), promote apoptosis, and facilitate phagocytosis by macrophages (Sugimoto et al., 2017), and is associated with NF-κB signaling pathway, serine/threonine protein kinase (Akt), and mitogen-activated protein kinase (MAPK) (Purvis et al., 2018). The above suggests that TMP is likely to participate in the body’s oxidative stress, immunoinflammation, drug metabolism, cartilage and bone metabolism through its action on ESR1, CAT, C3, CYP3A4, CYP2C9, and ANXA1, and thus play a role in treating OA.

### 4.3 Analysis of signaling pathways associated with TMP therapy for OA

From KEGG enrichment analysis, we found that neutrophil extracellular trap formation is an important signaling pathway for TMP to treat OA. Neutrophil extracellular traps (NETs) are large meshworks composed of DNA, histones, and granule proteins released by neutrophils (Brinkmann et al., 2004), which can trap and kill pathogens and induce thrombosis (Heestermans et al., 2016). In the microcirculation within the bone, thrombus can cause venous stasis and form intraosseous hypertension, which in turn alters the structure and function of the subchondral bone and articular cartilage tissues, induces an inflammatory response in the synovial membrane, disrupts the dynamic equilibrium between synthesis and degradation of cartilage matrix, and induces biomechanical alterations of articular cartilage, leading to degenerative changes in articular cartilage (Cui et al., 1992). Therefore, we believe that inhibiting the release of NETs or effectively improving stasis in OA patients may be one of the effective methods to delay the degeneration of articular cartilage. This animal experiment showed that knee joint effusion was significantly reduced in the TMP group compared with the OA group (*p* < 0.05). KEGG results also showed that some of the potential core target proteins of TMP, such as C3, HDAC8, MPO, ELANE, HDAC7, etc., were enriched in the signaling pathway of NET formation. The above suggests that TMP is likely to affect the NETs signaling pathway by acting on the key targets such as C3 and MPO, thus exerting the effects of anti-thrombosis, improving the joint microenvironment, and lowering the intraosseous pressure in the treatment of OA.

We found that the actin cytoskeleton regulatory signaling pathway is also one of the critical signaling pathways for TMP treatment of OA. The regulatory signaling pathway of the actin cytoskeleton is closely related to endothelial cell (EC) permeability.

When the EC actin skeleton changes, it will lead to EC contraction and increased permeability, leading to massive plasma extravasation, resulting in tissue edema and interstitial fluid accumulation (Wójciak-Stothard et al., 2001). This suggests that inhibiting the alteration of EC actin skeleton may be one of the effective methods to reduce joint effusion in OA. In addition, the regulatory signaling pathway of the actin cytoskeleton is also closely related to the phagocytosis of macrophages and the formation of immune synapses in T cells (Liu et al., 2020). Macrophages can phagocytose foreign particulate matter and pathogenic microorganisms. Actin can be involved in the regulation of phagocytosis in macrophages. When the dynamic changes of the macrophage actin cytoskeleton are inhibited, the phagocytosis function can be reduced [35]. T-cell immune synapse (IS) is a specific inter-cellular connectivity structure. Actin remodeling is involved in forming T-cell IS, which enhances the T-cell responses and affects the killing function of cytotoxic T lymphocytes (Na et al., 2015; Ritter et al., 2017). If actin remodeling is inhibited, T-cell responses can be reduced. This study showed that the potential core targets of TMP, such as BDKRB2, MSN, F2, and SLC9A1, were all enriched in the regulatory signal pathway of the actin cytoskeleton and participated in regulating the actin cytoskeleton. Animal experiments also showed that knee joint effusion was significantly reduced in the TMP group (*p* < 0.05). The above suggests that TMP is likely to affect the regulatory signaling pathway of the actin cytoskeleton by acting on the core target proteins, such as F2 and CDC42, and then play the roles of decreasing EC permeability, inhibiting the immune function of macrophages and T cells, and reducing the degree of joint effusion and inflammation in OA.

Based on KEGG results, we found that complement and coagulation cascade signaling pathways are also crucial for TMP treatment of OA. The coagulation cascade reaction consists of a series of enzymatic reactions that form a thrombus and stop bleeding, preventing bleeding after vessel rupture (Chen et al., 2012). However, if the coagulation cascade reaction is overactivated or abnormal, it may lead to thrombosis and thromboembolism (AlKoussa et al., 2022). Therefore, preventing overactivation of the coagulation cascade reaction is essential to prevent the progression of OA. Complement plays a vital role in the immune defense of the body. Some studies have shown that the expression of complement proteins is increased in both synovial tissue and synovial fluid in OA. At the same time, the membrane attack complex (MAC) content is positively correlated with the degree of synovial inflammation (Corvetta et al., 1992). In addition, chondrocytes also secrete complement proteins in response to IL-1β and TNF-α stimulation, promoting OA inflammation (Struglics et al., 2016). This suggests that targeted blockade of the complement system can effectively prevent OA progression and reduce OA inflammation. The results of this study showed that the essential target proteins of TMP, C3, BDKRB2, F2, and SERPINA5, were mainly enriched in the complement and coagulation cascade signaling pathway, and the molecular docking simulation also showed that TMP could be firmly bound to C3 and F2. The above suggests that TMP may influence the complement and coagulation cascade signaling pathways through essential target proteins, such as C3 and F2, and then regulate inflammation and thrombosis in OA.

### 4.4 The significance of clinical practice transformation of TMP therapy for OA

This experiment shows that TMP can hinder the progression of early arthritis, repair cartilage damage, and have a significant therapeutic effect on osteoarthritis. The screening of key therapeutic targets and treatment pathways for the treatment of osteoarthritis with TMP in this study has significant guiding significance for future clinical practice and greatly promotes the emergence of new methods for clinical treatment of osteoarthritis.

## 5 Conclusion

In summary, this study analyzed TMP’s targets, biological processes, related pathways, and effectiveness in treating OA using network pharmacology, molecular docking techniques, and animal experiments. TMP is effective in treating OA with multi-target and multi-pathway interactions. ESR1, CAT, C3, CYP3A4, CYP2C9, and ANXA1 may be potential targets for TMP treatment of OA. These key targets can affect OA by influencing endothelial cell permeability, microcirculation around joints, generation of NETs, activation of the complement system and coagulation pathway, regulation of immune function of macrophages and T cells, and biochemical metabolism of substances *in vivo*. The molecular mechanism mainly involves the formation of neutrophil extracellular trap, regulation of the actin cytoskeleton, complement and coagulation cascades, etc. It has potential significant implications for the development of new treatment strategies and personalized therapies in clinical practice.

## Data Availability

The original contributions presented in the study are included in the article/supplementary material, further inquiries can be directed to the corresponding authors.
